# Opposite roles of MAPKKK17 and MAPKKK21 against *Tetranychus urticae* in Arabidopsis

**DOI:** 10.3389/fpls.2022.1038866

**Published:** 2022-12-07

**Authors:** Gara Romero-Hernandez, Manuel Martinez

**Affiliations:** ^1^ Centro de Biotecnología y Genómica de Plantas (CBGP), Universidad Politécnica de Madrid (UPM)- Instituto Nacional de Investigación y Tecnología Agraria y Alimentaria (INIA)/CSIC, Madrid, Spain; ^2^ Departamento de Biotecnología-Biología Vegetal, Escuela Técnica Superior de Ingeniería Agronómica, Alimentaria y de Biosistemas, UPM, Madrid, Spain

**Keywords:** Arabidopsis, herbivore, MAP kinase, plant defense, signaling, *Tetranychus urticae*

## Abstract

After recognizing a biotic stress, plants activate signalling pathways to fight against the attack. Typically, these signalling pathways involve the activation of phosphorylation cascades mediated by Mitogen-Activated Protein Kinases (MAPKs). In the *Arabidopsis thaliana*-*Tetranychus urticae* plant-herbivore model, several Arabidopsis MAP kinases are induced by the mite attack. In this study, we demonstrate the participation of the MEKK-like kinases MAPKKK17 and MAPKKK21. Leaf damage caused by the mite was assessed using T-DNA insertion lines. Differential levels of damage were found when the expression of *MAPKKK17* was increased or reduced. In contrast, reduced expression of *MAPKKK21* resulted in less damage caused by the mite. Whereas the expression of several genes associated with hormonal responses did not suffer significant variations in the T-DNA insertion lines, the expression of one of these kinases depends on the expression of the other one. In addition, *MAPKKK17* and *MAPKKK21* are coexpressed with different sets of genes and encode proteins with low similarity in the C-terminal region. Overall, our results demonstrate that MAPKKK17 and MAPKKK21 have opposite roles. MAPKKK17 and MAPKKK21 act as positive and negative regulators, respectively, on the plant response. The induction of *MAPKKK17* and *MAPKKK21* after mite infestation would be integrated into the bulk of signalling pathways activated to balance the response of the plant to a biotic stress.

## Introduction

In nature, plants face numerous abiotic and biotic stresses. Regarding biotic attacks, the induction of plant defences is initiated by the activation of receptors that perceive conserved molecular patterns or specific molecules of attackers. In this recognition, there are two branches. One involves the use of transmembrane pattern recognition receptors (PRRs) that respond to microbe-, herbivore- or damaged-associated molecular patterns (MAMPs, HAMPs, or DAMPs), triggering an ordered sequence of molecular events called PAMP-triggered immunity (PTI) (Jones and Dangl, 2006; Zipfel, 2014; Santamaria et al., 2018a). The other branch encompasses the use of intracellular receptors that identify pathogen virulence molecules, known as effectors, that activate the effector-triggered immunity (ETI), an amplified response of the PTI (Tsuda and Katagiri, 2010). Upon recognition, a complex signalling process is triggered, which includes the rapid activation of specific ion channels, the production of reactive oxygen/nitrogen species (ROS/RNS), and the induction of specific cascades involving protein kinases and calcium-sensor proteins.

Typically, signalling pathways in plants involve the activation of a group of kinases named Mitogen-Activated Protein Kinases (MAPKs) (Thulasi Devendrakumar et al., 2018; Zhang et al., 2018; Zhang and Zhang, 2022). There are three MAPK groups: MAPKKKs (also named MAP3K or MEKK), MAPKKs (MAP2K, MKK, MEK), and MAPKs (or MPK). These proteins participate in a cascade when a stimulus is detected, suffering and causing post-translational phosphorylation (Krysan and Colcombet, 2018). The first protein activated by phosphorylation is a MAPKKK. Then, this protein phosphorylates two Ser/Thr residues separated by 5 amino acids in the phosphorylation loop of a MAPKK. Finally, activated MAPKK phosphorylates a MAPK in a loop containing Thr and Tyr residues separated by an amino acid (Jagodzik et al., 2018). In *Arabidopsis*, the repertoire of MAPKs consists of 20 MAPKs, 10 MAPKKs, and 80 MAPKKKs, the last group composed of 21 MEKK-like, 11 ZIK, and 48 Raf-like members (Jonak et al., 2002). These uneven numbers suggest that MAPK cascades are not linear, the same protein can be activated by several stimuli and could phosphorylate different MAPKs in the next step. To date, four cascades have been associated with biotic stresses in Arabidopsis, MAPKKK3/5/MEKK1-MKK4/5-MPK3/6; MEKK1-MKK1/2-MPK4; MAPKKK14-MKK3-MPK1/2/7; MAPKKK?-MKK9-MPK3/6 (Krysan and Colcombet, 2018; Lin et al., 2021). These cascades are typically based on responses to pathogens and involve the well-known MPK3, MPK4, and MPK6, controlled by less characterized MAP3K/MAP2K modules.

In a previous analysis, five MAP3Ks, *MAPKKK14*, *17*, *18*, *19*, and *21*; the MAP2K *MKK9*; and the MAPK *MPK11* were identified as commonly regulated by herbivory in Arabidopsis (Romero-Hernandez and Martinez, 2022). However, the direct participation of most of them in response to herbivores has not been reported yet, with the only exception of *MAPKKK14*, which was induced in response to the increased levels of jasmonic acid (JA) produced after wound treatment (Sözen et al., 2020). As MAPKKK14 activates the MKK3-MPK1/2/7 module and *mkk3* mutant plants are more susceptible to herbivory from larvae of *Spodoptera littoralis*, a role for the MAPKKK14 could be suggested to counteract insect feeding (Sözen et al., 2020). MAPKKK17 and 18 are also induced by wounding and are able to activate the MKK3-MPK1/2/7 module (Danquah et al., 2015; Sözen et al., 2020), which suggests a similar participation of MAPKKK17 and 18 in herbivore-related modules. In addition, MAPKKK17 and 18 are induced by ABA and affect ABA sensitivity (Danquah et al., 2015; Mitula et al., 2015; Choi et al., 2017). Notably, the relevance of MAP kinase cascades in plant responses to herbivory has been highlighted in other plant-herbivore models (Hettenhausen et al., 2015). For example, in *Nicotiana attenuata* and tomato plants, the participation of several MAPKs has been demonstrated in the response to the lepidopteran *Manduca sexta* (Kandoth et al., 2007; Wu et al., 2007; Hettenhausen et al., 2013). Likewise, several MAPKs modulate rice response to the herbivores *Niloparvata lugens* and *Chilo suppressalis* (Wang et al., 2013; Liu et al., 2018; Li et al., 2019; Zhou et al., 2019b).

Until now, the most useful model herbivore for Arabidopsis studies is the polyphagous mite *Tetranychus urticae* Koch (Tetranychidae) (Santamaria et al., 2020). To fight against mite attack, plants have evolved multiple constitutive and inducible barriers as direct defence mechanisms. After mite perception, triggered signalling pathways lead to the induction of several defence genes. Some of the genes involved in this defensive mechanism have been characterized, and their role against *T. urticae* has been proved. In the initial steps, the role of several genes in regulating ROS homeostasis in the Arabidopsis response to *T. urticae* infestation has been demonstrated (Santamaria et al., 2017; Santamaria et al., 2018b). Besides, a two-domain protein consisting of a Toll/Interleukin-1 receptor domain and a lectin domain encoded by the *PP2-A5* gene would be involved in a transcriptional reprogramming that altered plant defences against *T. urticae* mediated by strict regulation of the hormonal crosstalk between JA and SA mite-induced pathways (Zhurov et al., 2014; Santamaria et al., 2019). Regarding final defences, the role in mite-infested Arabidopsis plants has been reported for two genes encoding Kunitz trypsin inhibitors, several genes involved in the production of glucosinolates, and a gene encoding a hydroxynitrile lyase (*HNL*) involved in the production of free cyanide (Arnaiz et al., 2018; Arnaiz et al., 2022). Furthermore, using a transcriptomic meta-analysis typical common responses to herbivory were found, such as jasmonate signalling or glucosinolate biosynthesis, as well as particularities in the Arabidopsis-*T. urticae* interaction leading to the biosynthesis of anthocyanins and tocopherols (Garcia et al., 2021; Santamaria et al., 2021; Widemann et al., 2021).

In this study, we demonstrate the participation of the MEKK-like kinases MAPKKK17 and MAPKKK21 in the defence of the Arabidopsis plant against *T. urticae* and their opposite role in this process. Whereas MAPKKK17 acts as a positive regulator, MAPKKK21 has a negative effect on the plant response.

## Materials and methods

### Plant growth conditions

*Arabidopsis thaliana* plants, ecotype Col-0, were used as wild-type controls (WT). Arabidopsis T-DNA insertion lines (SALK_080309, SALK_137069, SALK_149019, SALK_018714, SALK_018804, and SALK_049352, named *mapkkk17_1*, *mapkkk17_2*, *mapkkk21_1*, *mapkkk21_2, mkk4_1, and mpk11_1*, respectively) were obtained from the Arabidopsis Biological Resource Centre, through the European Arabidopsis Stock Centre. For soil growth, seeds were surface sterilized with 70% (V/V) ethanol for 2 min, incubated in a solution containing 5% (V/V) SDS and 5% (V/V) NaClO for 12 min, and finally washed five times with sterilized deionized distilled H_2_O. Then, they were sowed in peat moss and vermiculite (2:1 v/v) and vernalized in the dark for 5 days at 4°C. After that, the plants grew in a growth chamber (Panasonic MLR-352-PE) at 23°C ± 1°C, >70% humidity and a 16-8h day/night photoperiod. For *in vitro* growth, seeds were sterilized as explained above and placed in plates containing MS1/2 medium (1% sucrose, 0.22% Murashige and Skoog with vitamins, 0.75% agar, adjusted to pH 5.6-5.9 with KOH). Germination chamber parameters were 21°C, 16-8h day/night photoperiod.

### Mite maintenance

A colony of *Tetranychus urticae* London strain (Acari: Tetranychidae), provided by Dr. Miodrag Grbic (UWO, Canada), was reared in beans (*Phaseolus vulgaris*) and kept in growth chambers (Sanyo MLR-351-H) at 25°C ± 1°C, >70% relative humidity and a 16h/8h day/night photoperiod.

### Nucleic acid analysis

The homozygous genotypes of T-DNA insertion lines were validated by conventional PCR. Through the Salk Institute website, primers were designed and used for this aim. Primer sequences are indicated in **Supplemental Table S1**. The genomic DNA used for conventional PCR was isolated from Arabidopsis T-DNA insertion and WT lines basically as described (Sambrook et al., 2001).

Real-time quantitative PCR (RT-qPCR) was used to validate the RNA-seq results, determine gene expression in T-DNA lines, and study the expression of other genes in the plant. For these analyses, 3-week-old Arabidopsis rosettes were sampled in non-infested plants or in plants at different times of mite infestation. Samples were frozen in liquid N_2_ and stored at -80°C until RNA isolation. Total RNA was extracted by the phenol/chloroform method, followed by precipitation with 8M LiCl (Oñate-Sánchez and Vicente-Carbajosa, 2008). Complementary DNAs (cDNAs) were synthesized from 2 μg of total RNA using the Revert AidTM H Minus First Strand cDNA Synthesis Kit (Fermentas) following the manufacturer’s instructions. The RT-qPCR conditions used were 40 cycles with 15 sec at 95°C, 1 min at 60°C, and 5 sec at 65°C using an SYBR Green Detection System (Roche) and the LightCycler^®^480 Software release 1.5.0 SP4 (Roche). Ubiquitin was used as the housekeeping gene and mRNA quantification was expressed as relative expression levels (2^-dCt^) (Livak and Schmittgen, 2001). Primer sequences are indicated in **Supplemental Table S1**.

### Plant damage determination

To quantify leaf damage, 3-week-old rosettes from T-DNA and WT lines were infested with 20 *T. urticae* female adults per plant for 4 days. Images for damage quantification were recovered using an HP Scanjet (HP Scanjet 5590 Digital Flatbed Scanner series). The total and damaged area of each rosette was measured using Adobe Photoshop CS software and analysed using Ilastik and Fiji, as previously described (Ojeda-Martinez et al., 2020). Chlorotic spots were quantified as leaf damage areas. Eight biological replicates from independent rosettes were used for each genotype.

For cell death quantification, leaf disks of 0.6 cm^2^ from 3 week-old plants were infested with 10 mites for 24h. Cell death was quantified by trypan blue staining, as described (Sanchez-Vallet et al., 2010). Images were taken with a Leica MZ10F lens and processed with the ImageJ program. Eight replicates were used for each genotype.

### Spider mite performance

To analyse the performance of spider mites feeding on WT and T-DNA lines, fecundity assays were performed. Spider mite female synchronization was conducted as described (Santamaria et al., 2019). Entire leaves detached from the WT and mutant plants were infested with 12 synchronized females each, and the number of eggs laid was counted after 36h of infestation. Eight biological replicates from independent rosettes were used for each genotype.

### Immunoblot analysis of MAPK activation

To perform Western blot assays, three-week-old rosettes of *A. thaliana* grown in MS1/2 medium were treated with 100 nM fls22 or infested with 20 adult female mites for 1h, 3h, and 24h. Eight rosettes per genotype were sampled and harvested in liquid nitrogen. MAPK activation was detected by immunoblot analysis of soluble proteins extracted from seedlings in a lysis buffer, using the Phospho-p44/42 MAPK (Erk1/2) (Thr202/Tyr204) antibody (Cell Signalling Technology).

### *In silico* analyses

The protein sequences of the MEKK-like Arabidopsis proteins were obtained using the Biomart tool in the EnsemblPlants database. The MEKK-like proteins were aligned using MUSCLE v3.8 (Edgar, 2004) and a phylogenetic tree was constructed using the maximum likelihood method in MEGA 11 (Tamura et al., 2021), using a BIONJ starting tree and applying bootstrap as a statistical test for branch support. The displayed pairwise sequence alignment was performed using the EMBOSS needle tool (Needleman and Wunsch, 1970). The predicted three-dimensional structures of the MAPKKK17 and MAPKKK21 proteins were obtained from the AlphaFold protein structure database (IDs AF-O80888-F1 and AF-Q6K1M3-F1, respectively). Spatial representations were visualized by the UCSF ChimeraX program (Pettersen et al., 2021).

Venn diagrams were performed using the InteractiVenn tool (Heberle et al., 2015). Gene enrichment analyses for biological processes were performed with the Fisher’s Exact test and the Benjamini-Hochberg false discovery rate (FDR) correction using the Panther 17.0 release (Bindea et al., 2009). NetworkAnalyst was chosen to construct gene molecular networks (Szklarczyk et al., 2019; Zhou et al., 2019a), using a confidence score higher than 400 and the minimum connected network option.

### Statistical analysis

Statistical analysis was performed using GraphPad Prism v8.01. To perform the proper statistical analysis, the normality and homoscedasticity of the data were previously analysed. When the data met both assumptions, one-way ANOVA followed by Student-Newman-Keuls multiple comparison test was run. Two-way ANOVA was performed in experiments where genotype (G) and mite treatment (T) were simultaneously analyzed and Student-Newman-Keuls multiple comparison test was used when the interaction (G×T) was significant.

## Results

### The expression of several MAP kinases is induced in response to *T. urticae*

**Figure 1:**
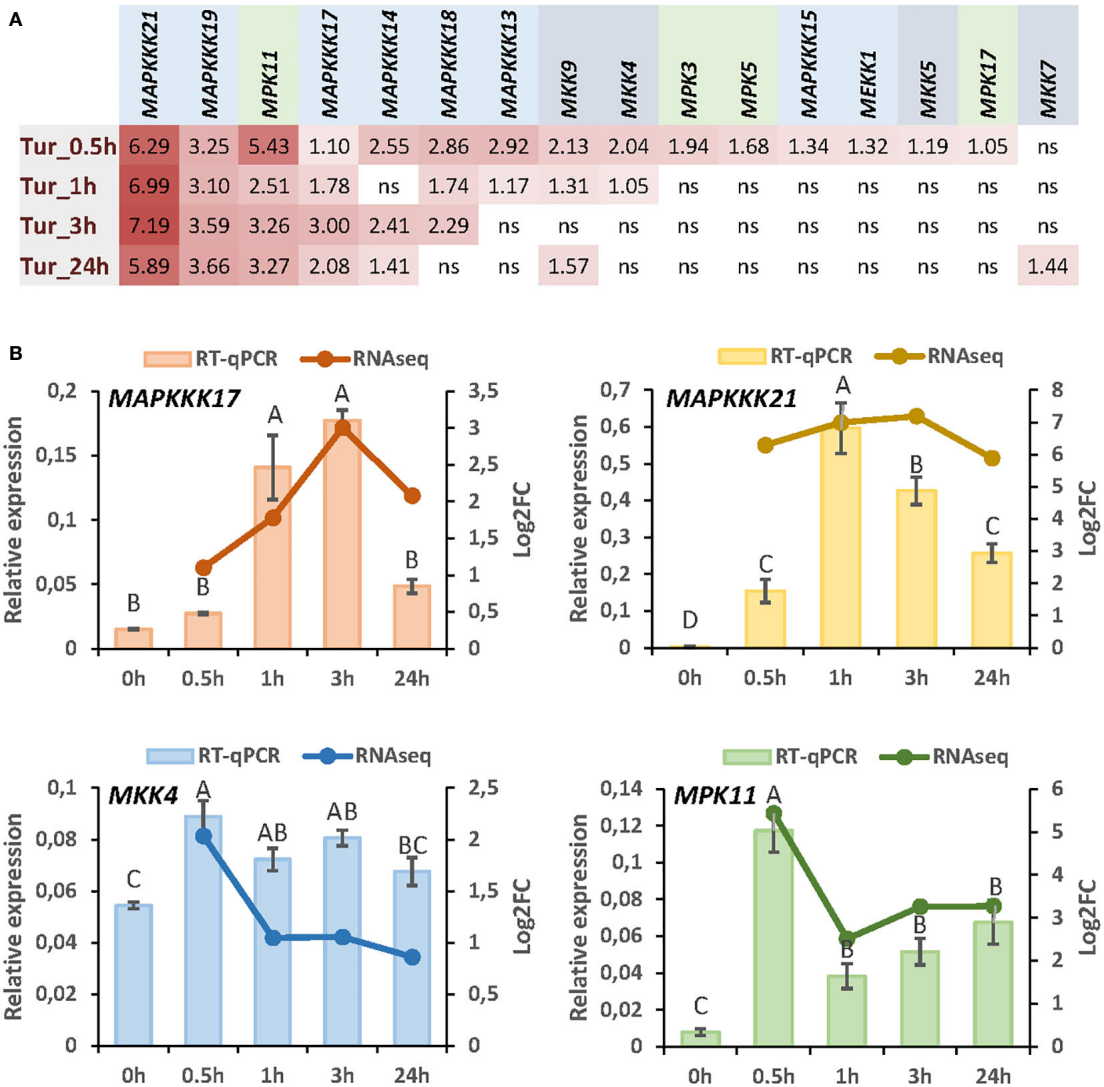
Expression of MAP kinases in response to *T. urticae* infestation. **(A)** Log2 fold change (FC) values of differentially expressed MAP kinases at 30min, 1h, 3h, and 24h of mite infestation. **(B)** Correlation between RNA-seq data and RT-qPCR validation for *MAPKKK17, MAPKKK21, MKK4, and MPK11* after 30min, 1h, 3h, and 24h of mite infestation. Data are means of three biological replicates. Different letters indicate significant differences (P<0.05, One-way ANOVA followed by Student-Newman-Keuls multiple comparisons test).

To understand the specificities in the role of MAP kinases regulated by herbivory in Arabidopsis, we focus on the response to a unique species. We select the spider mite *T. urticae* as we have previously reported an exhaustive transcriptomic analysis by RNA-seq at different times of mite infestation (Santamaria et al., 2021). From searches in the sets of genes differentially expressed after 30min, 1h, 3h, or 24h of infestation, 16 genes encoding MAPKs were found (**Figure 1A**). Among these genes, 8 encode MAPKKKs, 4 MAPKKs, and 4 MAPKs. All MAPKs except *MKK7* were induced after 30min and four of them, *MAPKKK21*, *MAPKKK19*, *MAPKKK17*, and *MPK11* were upregulated at the four time points. The MAPKKs that showed the highest induction were *MKK9* and *MKK4*. From these data, we selected some of the MAPK genes representing each step of the MAPK cascade that showed the highest induction after mite infestation. Induction of the four selected genes, *MAPKKK17*, *MAPKKK21*, *MKK4*, and *MPK11*, was corroborated by RT-qPCR analyses (**Figure 1B**).

### *MAPKKK17* and *MAPKKK21* are involved in Arabidopsis defence against *T. urticae*

**Figure 2:**
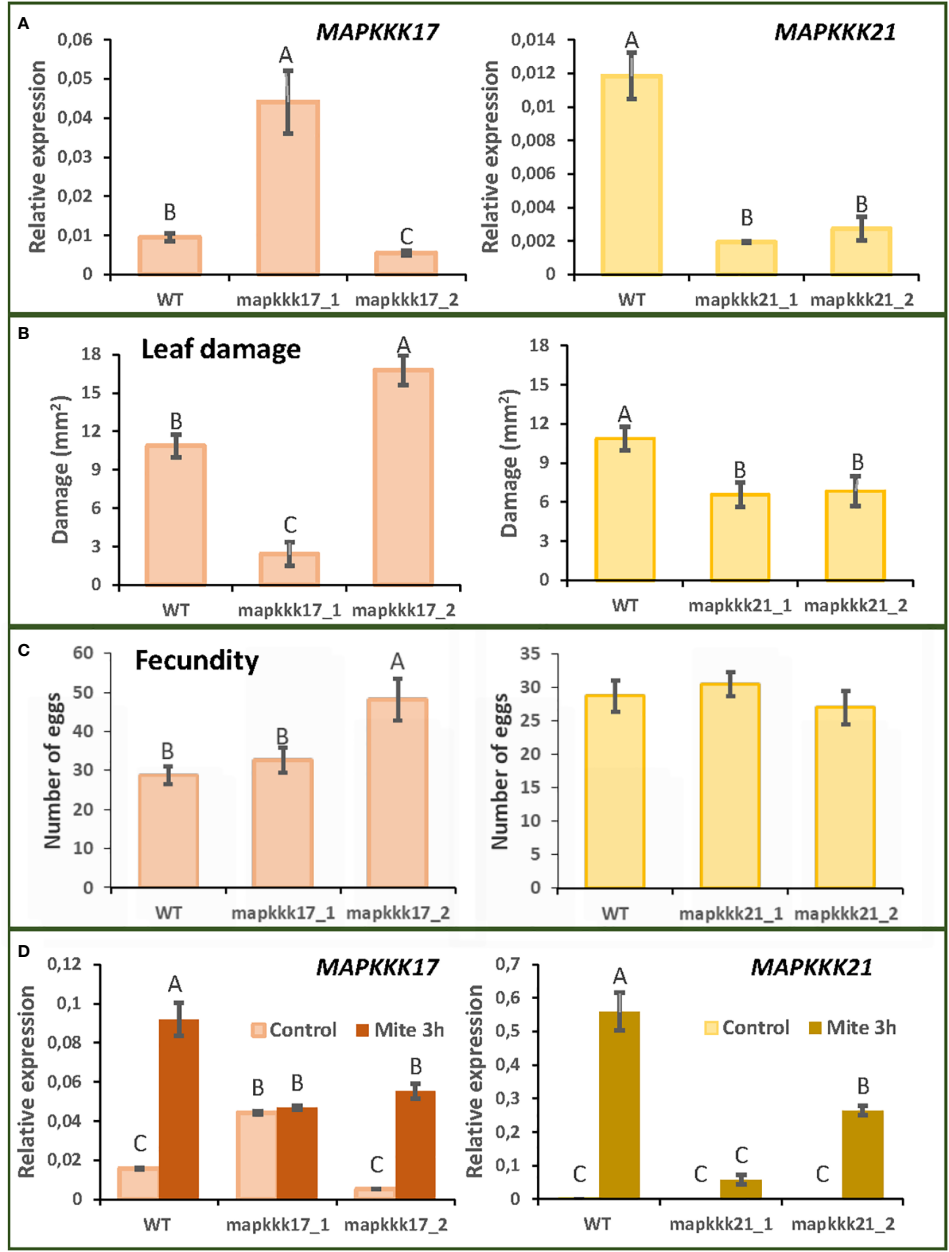
Plant damage and mite performance after feeding on Arabidopsis WT and T-DNA insertion lines for *MAPKKK17* and *MAPKKK21*. **(A)** Relative expression levels of *MAPKKK17* and *MAPKKK21* in WT and T-DNA inserted lines. Data are means ± SE of three biological replicates. **(B)** Foliar damaged area quantified after 4 d of mite infestation. Data are mean ± SE of eight replicates. **(C)** Effects on *T. urticae* fecundity quantified as the number of eggs 36 h after infestation with synchronized mite females. Data are mean ± SE of eight replicates. **(D)** Relative expression levels of *MAPKKK17* and *MAPKKK21* in WT and T-DNA inserted lines after 3h of mite infestation. Data are means ± SE of three biological replicates. Different letters indicate significant differences (P<0.05, One-way ANOVA followed by Student-Newman-Keuls multiple comparisons test).

T-DNA insertion lines were chosen for each selected MAPK to study the effect of these proteins. Two T-DNA insertion lines were selected for *MAPKKK17* and *MAPKKK21* and the only available T-DNA insertion lines for *MPKK4* and *MPK11*. The mutant lines of *MAPKKK17*, *mapkkk17_1*, and *mapkkk17_2*, have their insertions located after the kinase domain (**Supplementary Figure 1**). Whereas the expression of the *MAPKKK17* gene in *mapkkk17_1* was higher than in the WT plants, it was lower in *mapkkk17_2* (**Figure 2A**). In the case of *MAPKKK21*, the mutant lines *mapkkk21_1* and *mapkkk21_2* have the insertion in the 5’UTR and the 3’UTR, respectively (**Supplementary Figure 1**). Both lines had a lower expression of the *MAPKKK21* gene than WT plants (**Figure 2A**). The mutant lines of *MPKK4* and *MAPK11* contained the T-DNA inserted in the 3’UTR and in the kinase domain, respectively, being *mpkk4_1* a knockdown line and *mpk11_1* a knockout line (**Supplementary Figures 1** and **Supplementary Figure 2**).

To analyse the consequences of these insertions on Arabidopsis defence against *T. urticae*, leaf damage assays were performed. Whereas the *mapkkk17_1* line, overexpressing *MAPKKK17*, showed minor leaf damage caused by the mite after 4 days of infestation, the *mapkkk17_2* silenced line showed higher damage (**Figure 2B**). In the case of the two *MAPKKK21* mutant lines, the damage caused by mite feeding was lower than that observed in WT plants (**Figure 2B**). Variations in leaf damage were not found for the only *MKK4* and *MAPK11* T-DNA insertion lines (**Supplementary Figure 2**). These results led us to focus on the *MAPKKK* genes with an observed phenotype after mite infestation.

To evaluate mite performance, a fecundity test was performed with the mutant lines of *MAPKKK17* and *MAPKKK21*. Only the mutant line of *MAPKKK17*, *mapkkk17_2*, showed differences in the number of eggs laid, compared to the WT plants (**Figure 2C**). To verify the expression of *MAPKKK17* and *MAPKKK21* in the mutant lines after mite infestation, a feeding assay with *T. urticae* during 3h of infestation was performed (**Figure 2D**). In the case of WT plants, the expression of *MAPKKK17* and *MAPKKK21* genes increased as expected. In *MAPKKK17* and *MAPKKK21* knockdown lines, their expression increased after mite infestation but was lower than in WT. In the line overexpressing *MAPKKK17*, no differences in its expression were found after mite infestation. In addition, cell death was quantified to compare mite feeding with leaf damage. After 24h of infestation, the *mapkkk17_1* line showed higher trypan blue staining than WT plants. For *MAPKKK21*, the *mapkkk21_1* line had significantly less cell death area than WT (**Supplementary Figure 3**).

### *MAPKKK17* and *MAPKKK21* have evolutionary differences and are co-expressed with different sets of genes

**Figure 3:**
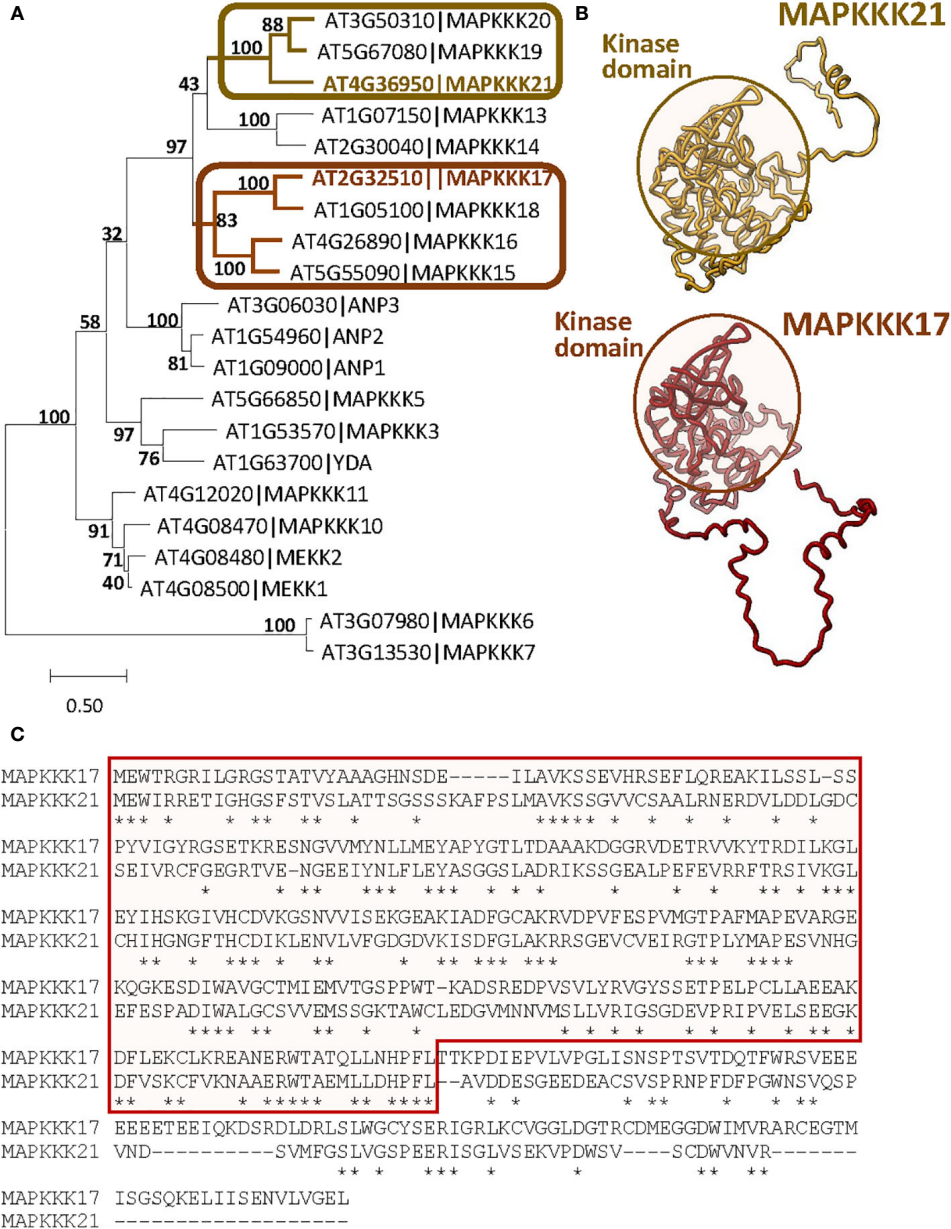
Sequence-structure comparison of MAPKKK17 and MAPKKK21. **(A)** Phylogenetic tree constructed by the maximum likelihood method with the 21 MEKK-like proteins identified in Arabidopsis. Bootstrap values are shown. Branches containing MAPKKK17 and MAPKKK21 are highlighted. **(B)** AlphaFold models predicted for MAPKKK17 and MAPKKK21. Kinase domains are circled. **(C)** Pairwise alignment of the amino acid sequences of MAPKKK17 and MAPKKK21. The kinase domain is squared. Asterisks identified identical residues.

The differences in the effects of MAPKKK17 and MAPKKK21 could be related to variations in the sequence and structure produced during evolution. To investigate the evolutionary distance between both proteins, a phylogenetic tree was constructed using the 21 MEKK-like proteins identified in Arabidopsis (**Figure 3A**). Although both proteins are in a clade with other 7 proteins, MAPKKK17 and MAPKKK21 are included in different branches. Whereas MAPKKK21 is grouped with MAPKKK19 and 20, MAPKKK17 is closer to MAPKKK15, 16, and 18. A comparison between the tridimensional structures predicted by AlphaFold showed a very conserved part that included the kinase domain, and different coiled structures (**Figure 3B**). These differences reflect high variations in the amino acid sequence of the C-terminal region, which is longer in MAPKKK17 than in MAPKKK21 (**Figure 3C**).

**Figure 4:**
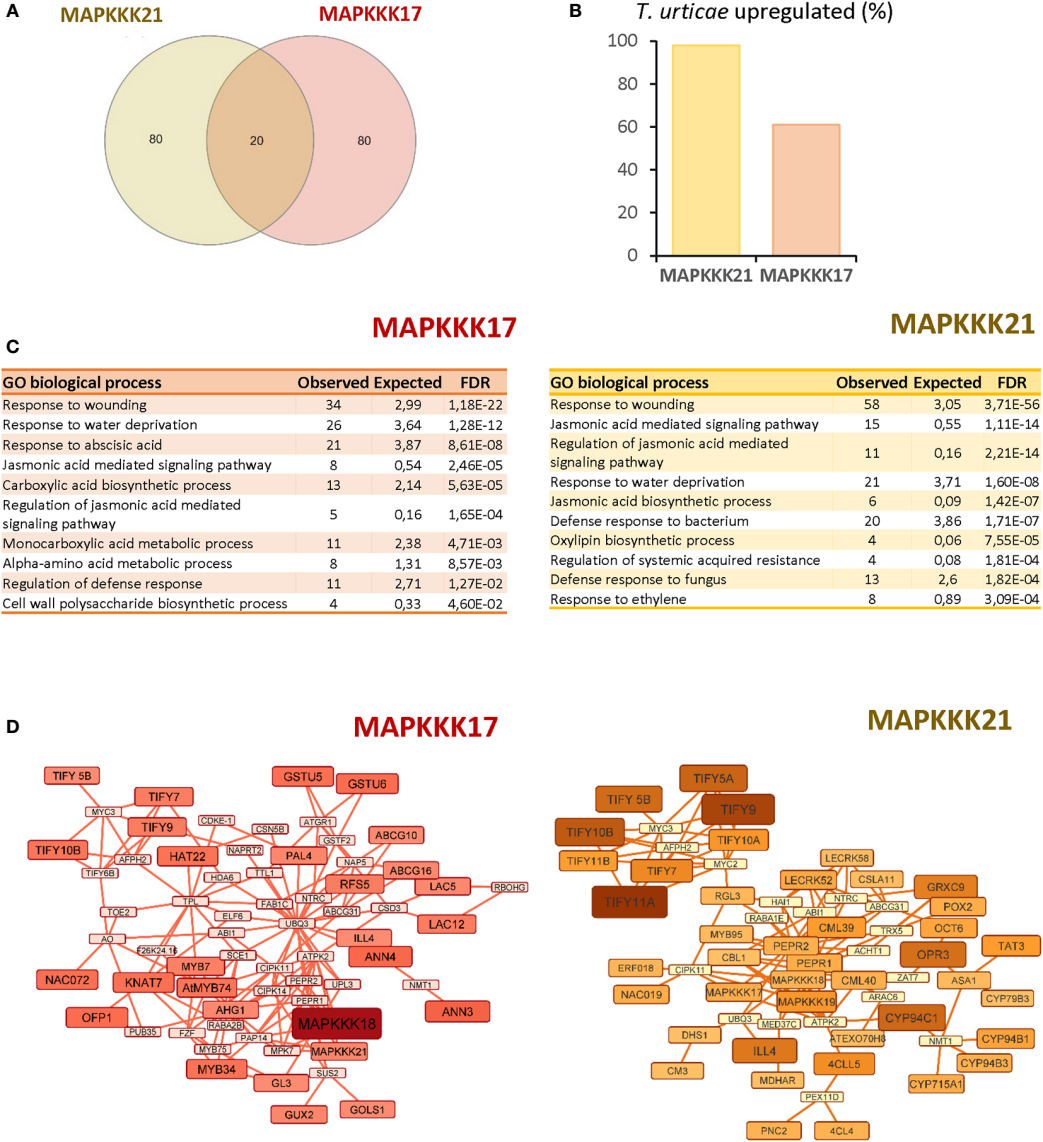
Comparison of the 100 most correlated genes to *MAPKKK17* and *MAPKKK21*. **(A)** Venn diagram showing the numbers of shared and specific genes correlated to *MAPKKK17* and *MAPKKK21*. **(B)** Percentage of the genes that were identified as differentially expressed in the RNA-seq analysis after mite infestation. **(C)** Lists of the 10 most significantly enriched biological processes for each correlated list of genes. **(D)** Networks connecting the most correlated genes to *MAPKKK17* and *MAPKKK21*. Larger node sizes and darker colors identified the genes with the highest correlation coefficients.

To obtain information on the functional consequences of the sequence-structure variations, the 100 genes with the highest expression correlation with each MAPK were obtained from the ATTED II database (**Supplementary Dataset 1**). Of these 100 genes, only 20 genes were shared on the two lists (**Figure 4A**). Searches in the differentially expressed sets of genes from the previous RNA-seq analysis of the Arabidopsis response to *T. urticae* identified 98 of the *MAPKKK21* correlated genes, but only 60 of the *MAPKKK17* gene list (**Figure 4B**). Gene ontology analyses identified an enrichment of genes involved in various biological processes in both sets of co-expressed genes (**Figure 4C**). The shared enriched processes included those related to defence, such as “Response to wounding” or “Jasmonic acid signalling pathway”, which were more significantly enriched in the *MAPKKK21* gene list. In addition, some biological processes were differentially enriched. Some examples are “Cell wall polysaccharide biosynthetic process” and “Response to abscisic acid” in the list of *MAPKKK17*, and “Jasmonic acid biosynthetic process” and “Response to ethylene” in the list of *MAPKKK21*. Similarities and differences were reinforced in the networks constructed using the NetworkAnalyst tool (**Figure 4D**). In the network using the *MAPKKK21* correlated genes, several genes involved in the negative regulation of jasmonic acid signalling were found, such as several *TIFY* regulators and the *CYP94C1, CYP94B1, CYP94B3*, and *ILL4* genes involved in the catabolism of active jasmonic acid. In the network for *MAPKKK17*, the relevance of genes related to abscisic acid (ABA) signalling and cell wall control is remarkable, including some annexins and laccases, and the transcription factors *HAT22*, *KNAT7*, and *MYB74*.

### Effect of mutant backgrounds on the expression of genes involved in hormonal pathways

**Figure 5:**
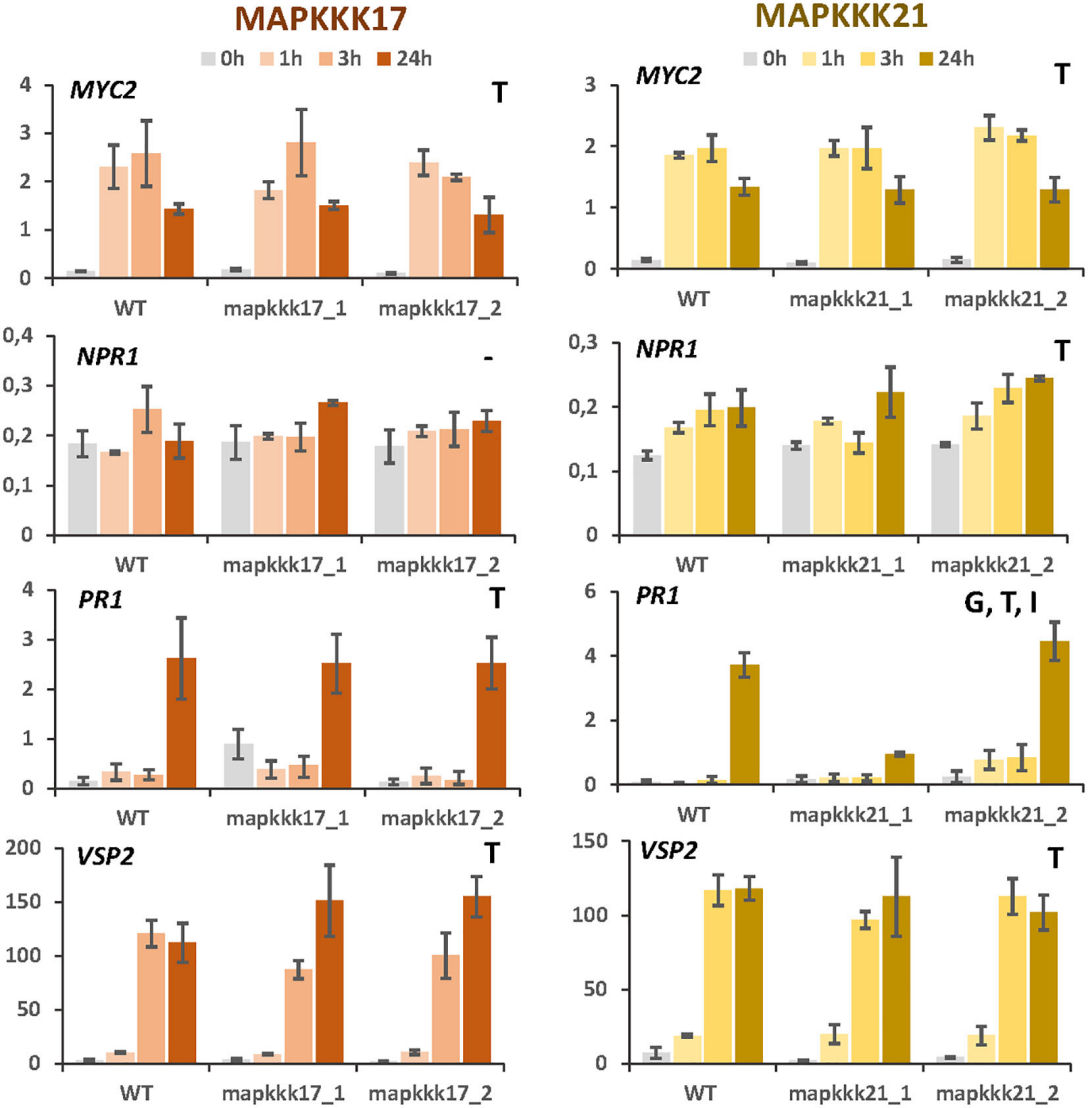
Relative expression of *MYC2*, *NPR1*, *PR1*, and *VSP2* genes in different Arabidopsis backgrounds. Gene expression was quantified by RT-qPCR after 1h, 3h, and 24h of mite feeding on WT and T-DNA mutant lines. Data are means ± SE of three biological replicates. Significant factors (SF) indicate whether the two independent factors, T (treatment with mites) and G (genotype), and/or their interaction, I (T×G), were statistically significant (Two-way ANOVA, P<0.05, followed by Student-Newman-Keuls multiple comparisons test).

The observed enrichment of pathways related to hormonal pathways associated with defence suggests a modulation caused by MAPKKK17 and MAPKKK21. To test this hypothesis, expression analyses of the genes encoding the transcription factors MYC2 and NPR1 and the final products VSP2 and PR1 were performed at different times of mite infestation in WT and mutant lines (**Figure 5**). The two genes involved in the jasmonate signalling pathway, *MYC2* and *VSP2*, were induced similarly after mite infestation in WT plants and in T-DNA insertion lines for *MAPKKK17* and *MAPKKK21*, without significant differences between genotypes. Similarly, the genes selected to check the salicylic acid (SA) pathway, *NPR1* and *PR1*, did not show relevant differences between genotypes, with the exception of *PR1*, which was significantly less induced at 24h in the *mpkkk21_1* mutant line. These results suggest a limited modulation of MAPKKK17 and MAPKKK21 on the expression of genes related to the JA and SA hormonal pathways.

### Effect of mutant backgrounds on the expression of MAP kinases

**Figure 6:**
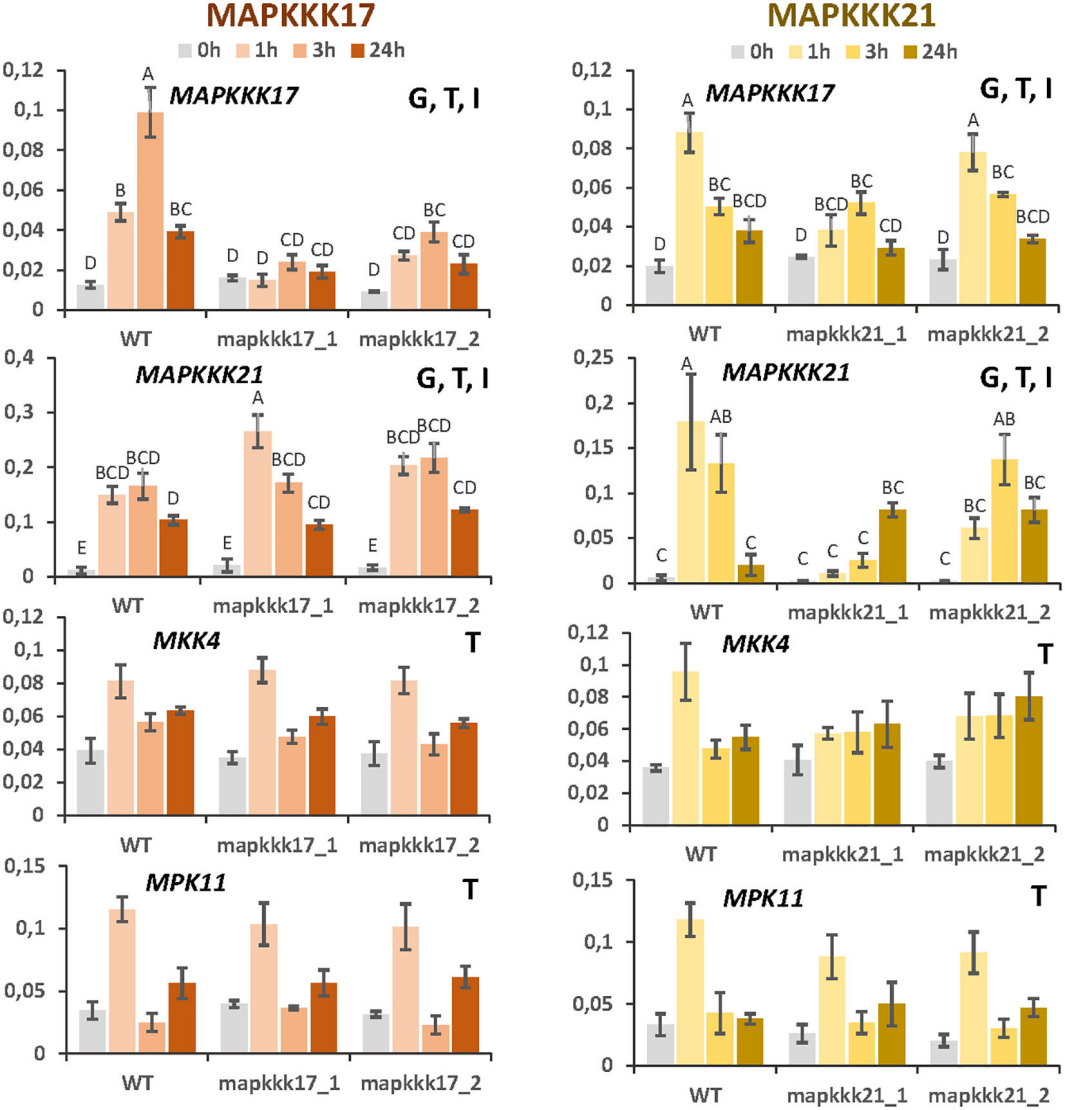
Relative expression of *MAPKKK17*, *MAPKKK21*, *MKK4*, and *MPK11* genes in different Arabidopsis backgrounds. Gene expression was quantified by RT-qPCR after 1h, 3h, and 24h of mite feeding on WT and T-DNA mutant lines. Data are means ± SE of three biological replicates. Significant factors (SF) indicate whether the two independent factors, T (treatment with mites) and G (genotype), and/or their interaction, I (T×G), were statistically significant (Two-way ANOVA, P<0.05, followed by Student-Newman-Keuls multiple comparisons test). Different letters indicate significant differences.

Changes in the expression of *MAPKKK17* and *MAPKKK21* due to the T-DNA insertion could affect the transcriptomic regulation of other MAP kinases. First, the expression of selected MAP kinases was checked in the mutant lines after spider mite infestation (**Figure 6**). As previously observed, *MAPKKK17* expression was not induced by the mite in the line overexpressing this gene. In the knock-down line, its expression increased but at lower levels than in WT plants. In addition, *MAPKKK21* expression was highly induced in the overexpressing *mapkkk17_1* line than in WT and *mapkkk17_2* line after an hour of mite infestation. The knockdown lines of *MAPKKK21* behaved differently. The expression of the *MAPKKK17* and *MAPKKK21* genes in *mpkkk21_1* was lower and suffered a delay in their induction than in WT plants. In *mpkkk21_2*, *MAPKKK17* expression behaved similarly to WT plants but the peak of *MAPKKK21* expression appeared later than in WT plants after mite infestation. In addition, the expression of the mite-induced *MKK4* and *MPK11* genes did not change in any of these T-DNA insertion lines compared to WT plants at any time after infestation (**Figure 6**).

The expression of the MAP kinases described as important for the plant response against pathogens was also monitored. The expression level of any of them, *MPK3*, *MPK4*, and *MPK6*, was affected when the expression of *MAPKKK17* or *MAPKKK21* was modified by the insertion of the T-DNA (**Supplementary Figure 4**). Furthermore, Western blot assays were performed to analyse the pattern of phosphorylated MAP kinases after mite infestation. Western blots showed an induction of the MPK4/11 band at different times of infestation compared to the non-infested control (**Supplementary Figure 5**). Slight differences were found for the bands associated with MPK3 and MPK6 kinases. When the patterns of phosphorylated MAP kinases were compared between mutant and WT plants, no obvious differences were observed after mite infestation.

## Discussion

Signalling is a key process to reaching an efficient response to any putative threat. Among the actors involved, MAP kinases play crucial roles as they can interact and phosphorylate other proteins, changing their functional status. Specificities in this process are expected to respond against individual threats. We had previously identified several MAP kinases involved in the three steps of the cascade with a putative predominant role in Arabidopsis against herbivory (Romero-Hernandez and Martinez, 2022). To find specific responses, we focus on changes in the expression of MAP kinases against the spider mite *T. urticae*. Notably, the most induced MAP kinases correspond to those identified as involved in a general response to herbivory, which were induced along mite infestation, from 30 min to 24h. Other MAP kinases were occasionally induced at lower levels, which suggests that they could have a minor effect on signalling. In Arabidopsis, the activated MAPK cascades described in response to pathogens typically involve the MAPKs MPK3, MPK4, and MPK6. MPK3/6 perform redundant functions upon *Botrytis cinerea* or flagellin treatment and trigger SA-dependent responses, which are negatively regulated by MPK4 (Brodersen et al., 2006; Bethke et al., 2009; Mao et al., 2011; Li et al., 2012; Zhang et al., 2012). Consistently with the previous identification as kinases no induced by herbivory, only MPK3 was slightly induced at 30 min of mite infestation and no changes in the intensity of bands corresponding to MPK3 and MPK6 activated proteins were found in Western blot assays. Besides, the band corresponding to the MPK4/11 kinases was more intense after mite infestation, probably due to the high activation of MPK11 associated with its induced expression. These results confirmed the particularities in the herbivore response.

MAP kinase cascades could be represented as a complex network of interactions converting an input signal into an output response. Redundancy and multifunctionality are critical concepts to understand their roles in the plant. Some examples are the kinase cascades that have been associated with biotic stresses in Arabidopsis, MAPKKK3/5/MEKK1-MKK4/5-MPK3/6; MEKK1-MKK1/2-MPK4; MAPKKK14-MKK3-MPK1/2/7; MAPKKK?-MKK9-MPK3/6 (Krysan and Colcombet, 2018; Lin et al., 2021). These cascades have multiple kinases involved in each step and share some of their components. This implies that no phenotypical effects could be observed when a kinase is silenced. In our analyses, silencing MKK4 or MPK11 kinases did not cause differences in the leaf damage caused by the mite. Contrarily, T-DNA insertions in *MAPKKK17* and *MAPKKK21* had consequences in mite feeding. Interestingly, opposite features were found. Silenced *MAPKKK17* and *MAPKKK21* lines were more and less susceptible to mite feeding, respectively. Furthermore, mite infestation caused more cell death in the overexpressing *MAPKKK17* line but less leaf damage. Whereas cell death has been directly associated with mite feeding, chlorotic spots are a symptom of plant damage caused by mite attack but are not an immediate consequence of mite feeding (Bensoussan et al., 2016).

The question here is to understand why two kinases highly induced after mite infestation have this antagonist role. As Arabidopsis defence against *T. urticae* depends on the activation of the JA and SA pathways (Zhurov et al., 2014; Santamaria et al., 2019), MAPKKK17 and 21 could be involved in the activation/repression of these pathways. However, the expression of several genes associated with JA and SA responses did not suffer extensive variations in the T-DNA insertion lines. Additional clues can be obtained from past experiments. After wounding treatment, the expression of *MAPKKK14*, *15*, *17*, *18*, and *19* were induced (Sözen et al., 2020). All of these kinases were also induced by *T. urticae* feeding. Wound-induced *MAPKKK14* was shown to activate the MKK3-MPK1/2/7 module. This module was associated with defence against insect feeding as *mkk3* mutant plants were more susceptible to herbivory from larvae of *S. littoralis* (Sözen et al., 2020). In addition, *MAPKKK17* and *18* are induced by ABA and activate the MKK3-MPK1/2/7/14 module, which is regulated by ABA (Danquah et al., 2015). These findings would support a role for this module in defence against *T. urticae*. However, any of the *MKK* and *MPK* kinases of the module were induced by mite feeding. Besides, previous information regarding *MAPKKK21* was not found in the literature.

As MAP kinases are involved in signalling pathways, the genes whose expression correlated with that of *MAPKKK17* and *21* could give us information on their participation in biological processes. Differences were clearly found. *MAPKKK21* is commonly expressed together with genes involved in plant defence, and almost all of these genes were also induced after mite infestation. On the contrary, whereas a set of genes coexpressed with *MAPKKK17* is also involved in plant defence and induced after mite infestation, groups of genes related to response to ABA or involved in the biosynthesis of the cell wall were found. Interestingly, the most correlated gene for *MAPKKK17* was *MAPKKK18*, and both genes were induced by ABA as previously mentioned (Danquah et al., 2015; Mitula et al., 2015). Therefore, MAPKKK17 could be defined as a multifunctional kinase induced by different stimuli and involved in the positive defence response of the plant against mite feeding.

The physiological roles of MAPKKK21 should be different. Strong correlation values with genes involved in plant defence suggest prioritized participation in signalling pathways involved in the response to biotic stimuli. Remarkably, silencing *MAPKKK21* lines were less susceptible to mite attack, suggesting a negative role in plant defence. Negative roles have been described for some other genes induced after mite attack. For example, several genes involved in the catabolism of the most bioactive JA form, jasmonoyl-isoleucine (JA-Ile), are upregulated after mite attack. These genes are included in the two metabolic pathways downstream of JA-Ile that have been described (Marquis et al., 2020). One includes the oxidation of JA-Ile mediated by CYP94C1, CYP94B1, and CYP94B3, to generate the inactive derivative 12-COOH-JA-Ile (Heitz et al., 2012; Koo et al., 2014). In the second pathway, JA-Ile is hydrolysed by IAR3/ILL4 to finally generate 12-OH-JA (Widemann et al., 2013; Zhang et al., 2016). Notably, the expression of all these genes is highly correlated with that of *MAPKKK21*. Likewise, JAZ/TIFY are negative regulatory proteins that directly interact with MYC2, leading to transcriptional repression of JA-responsive genes (Chini et al., 2007). Eight members of the JAZ/TIFY protein family are highly coexpressed with *MAPKKK21*.

Furthermore, the expression of *MAPKKK17* is conditioned by the level of expression of *MAPKKK21*, and vice versa, which supports the interconnection of both proteins in the signalling pathways leading to plant responses. Spatiotemporal specificities in the expression of activating proteins and their putative substrates have been postulated as major components contributing to signalling specificity (Zhang and Zhang, 2022). Besides, their roles should be conditioned by the ability of each MAP kinase to interact with other proteins. MAPKKK17 and 21 have a conserved kinase domain and a C-terminal disordered region with low similarity. This C-terminal region has been associated with specificities in the interactors of this group of kinases. Therefore, the different roles are further supported by their capacity to putatively interact with different proteins. These proteins should not be necessarily their activators or members of the MPKK family. The interaction of MAPKKK kinases with non-kinase proteins has already been described. For example, HEAT SHOCK PROTEIN 90 (HSP90) interacts with the Arabidopsis MAPKKK YDA during embryogenesis (Samakovli et al., 2020). In addition, *in vitro* interaction has been described for MAPKKK19 with PEN3, an ABC transporter involved in exporting antimicrobial metabolites (Campe et al., 2016). Likewise, MAPKKK20 interacts *in vitro* with several calmodulin-like proteins (Popescu et al., 2007), such as CAM1, recently described as a negative regulator of JA biosynthesis (Jiao et al., 2022). The biological implications of these interactions should be demonstrated *in vivo*. Furthermore, the magnitude and duration of MAP kinase signalling effect depend on their activation by upstream kinases and their inactivation by protein phosphatases (Schweighofer et al., 2007; Bartels et al., 2010; Ayatollahi et al., 2022).

In conclusion, the participation of MAPKKK17 and MAPKKK21 in the defence of the Arabidopsis plant against *T. urticae* has been demonstrated. Both kinases are relevant to achieving full defence but have different roles. MAPKKK17 is a signal activator that enhances defence when overexpressed previously to mite attack. MAPKKK21 is a negative regulator of plant defence as determined by the lower damage caused by the mite when *MAPKKK21* expression is lowered by T-DNA insertions. The induction of *MAPKKK21* after mite infestation would be integrated into the bulk of signalling pathways activated to balance the response of the plant to a biotic stress. Continuous activation of defence responses would have a deleterious effect on the development of the plant that is compensated by activation of pathways to stop responses. Further studies are needed to know which mechanisms are involved in the action of MAPKKK21.

## Data availability statement

The original contributions presented in the study are included in the article/**Supplementary Material**. Further inquiries can be directed to the corresponding author.

## Author contributions

MM conceived and designed the study. GR-H performed most of the experimental research. MM performed the bioinformatics analyses. All authors contributed to the article and approved the submitted version.

## Supplementary material

Supplementary Figure 1 Scheme of the position of the T-DNA insertions in the mutant lines.

Supplementary Figure 2 Relative expression levels of *MKK4* and *MPK11* in WT and T-DNA inserted lines. Data are means ± SE of three biological replicates. Foliar damaged area quantified after 4 d of mite infestation. Data are mean ± SE of eight replicates.

Supplementary Figure 3 Quantification of trypan blue staining after 24 h of mite infestation in Arabidopsis WT and T-DNA insertion lines for *MAPKKK17* and *MAPKKK21*. Data are mean ± SE of eight replicates. Different letters indicate significant differences (P<0.05, One-way ANOVA followed by Student-Newman-Keuls multiple comparisons test).

Supplementary Figure 4 Relative expression of *MPK3*, *MPK6*, and *MPK4* genes in different Arabidopsis backgrounds. Gene expression was quantified by RT-qPCR after 1h, 3h, and 24h of mite feeding on WT and T-DNA mutant lines. Data are means ± SE of three biological replicates. Significant factors (SF) indicate whether the two independent factors, T (treatment with mites) and G (genotype), and/or their interaction, I (T×G), were statistically significant (Two-way ANOVA, P<0.05, followed by Student-Newman-Keuls multiple comparisons test).

Supplementary Figure 5 Western blot analysis of MAPK activation after 1h, 3h, and 24h of mite treatment in WT and T-DNA mutant lines. Flagellin treatment was used as a control of band size for MPK3/4/6. Activated MAPKs were detected by immunoblots using α-p44/42-ERK antibody. Equal loading is indicated by the Ponceau S staining of Rubisco.

Supplementary Table 1 Sequences of the oligonucleotides used in this work.

Supplementary Data Sheet 1 Lists of 100 most significant coexpressed genes to *MAPKKK17* and *MAPKKK21*.
