# Supplementary figures and images for: Opposite roles of MAPKKK17 and MAPKKK21 against Tetranychus urticae in Arabidopsis
Source: Front Plant Sci. 2022 Dec 7;13:1038866. doi: 10.3389/fpls.2022.1038866 (PMC9768502; doi:10.3389/fpls.2022.1038866)

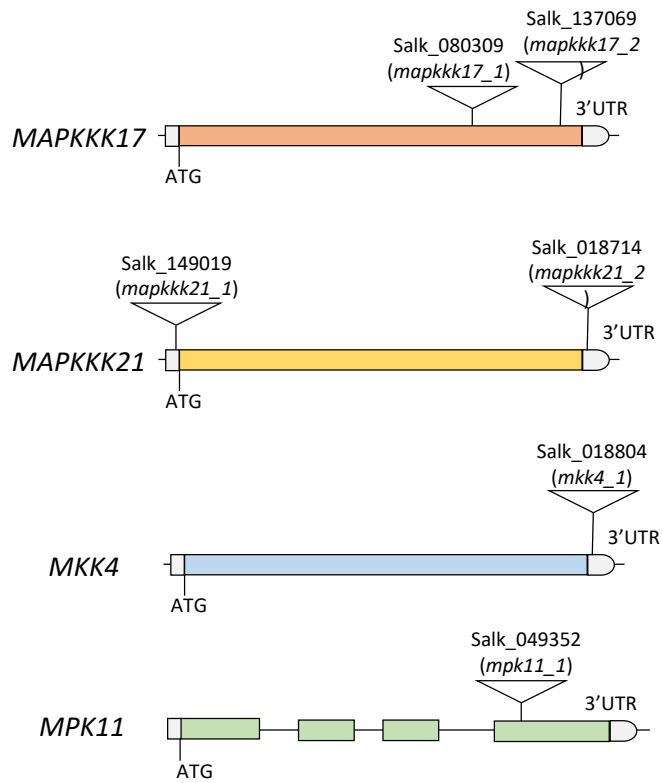

**Suppl. Figure 1.** Scheme of the position of the T-DNA insertions in the mutant lines.

Supplement: Supplementary Figure 1 — Scheme of the position of the T-DNA insertions in the mutant lines. [file Image_1.pdf]
