# Supplementary material for: Opposite roles of MAPKKK17 and MAPKKK21 against Tetranychus urticae in Arabidopsis
Source: Front Plant Sci. 2022 Dec 7;13:1038866. doi: 10.3389/fpls.2022.1038866 (PMC9768502; doi:10.3389/fpls.2022.1038866)
Supplement: Supplementary Figure 2 — Relative expression levels of MKK4 and MPK11 in WT and T-DNA inserted lines. Data are means ± SE of three biological replicates. Foliar damaged area quantified after 4 d of mite infestation. Data are mean ± SE of eight replicates. [file Image_2.pdf]

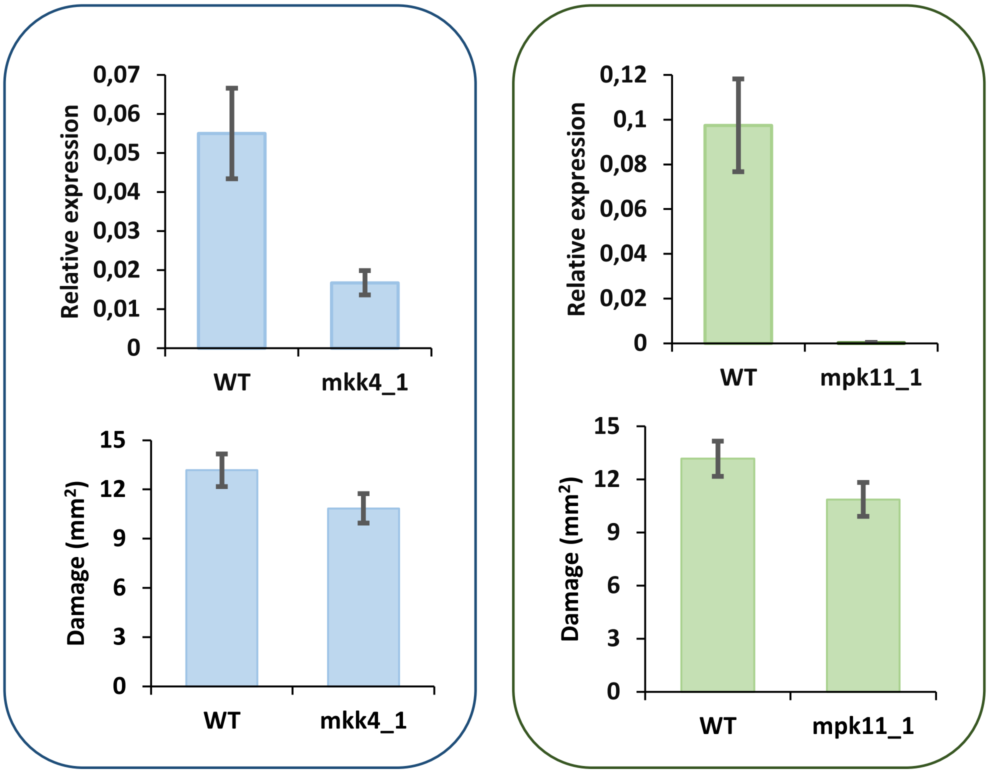

Suppl. Figure 2. Relative expression levels of *MKK4* and *MPK11* in WT and T-DNA inserted lines. Data are means ± SE of three biological replicates. Foliar damaged area quantified after 4 d of mite infestation. Data are mean ± SE of eight replicates.
