# Supplementary material for: Opposite roles of MAPKKK17 and MAPKKK21 against Tetranychus urticae in Arabidopsis
Source: Front Plant Sci. 2022 Dec 7;13:1038866. doi: 10.3389/fpls.2022.1038866 (PMC9768502; doi:10.3389/fpls.2022.1038866)
Supplement: Supplementary Figure 3 — Quantification of trypan blue staining after 24 h of mite infestation in Arabidopsis WT and T-DNA insertion lines for MAPKKK17 and MAPKKK21. Data are mean ± SE of eight replicates. Different letters indicate significant differences (P<0.05, One-way ANOVA followed by Student-Newman-Keuls multiple comparisons test). [file Image_3.pdf]

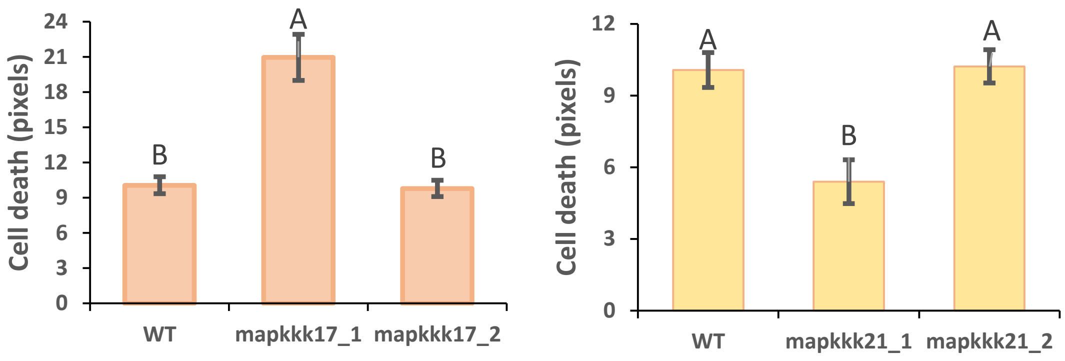

Suppl. Figure 3. Quantification of trypan blue staining after 24 h of mite infestation in Arabidopsis WT and T-DNA insertion lines for *MAPKKK17* and *MAPKKK21*. Data are mean ± SE of eight replicates. Different letters indicate significant differences (P<0.05, One-way ANOVA followed by Student-Newman-Keuls multiple comparisons test).
