# Supplementary material for: Opposite roles of MAPKKK17 and MAPKKK21 against Tetranychus urticae in Arabidopsis
Source: Front Plant Sci. 2022 Dec 7;13:1038866. doi: 10.3389/fpls.2022.1038866 (PMC9768502; doi:10.3389/fpls.2022.1038866)
Supplement: Supplementary Figure 4 — Relative expression of MPK3, MPK6, and MPK4 genes in different Arabidopsis backgrounds. Gene expression was quantified by RT-qPCR after 1h, 3h, and 24h of mite feeding on WT and T-DNA mutant lines. Data are means ± SE of three biological replicates. Significant factors (SF) indicate whether the two independent factors, T (treatment with mites) and G (genotype), and/or their interaction, I (T×G), were statistically significant (Two-way ANOVA, P<0.05, followed by Student-Newman-Keuls multiple comparisons test). [file Image_4.pdf]

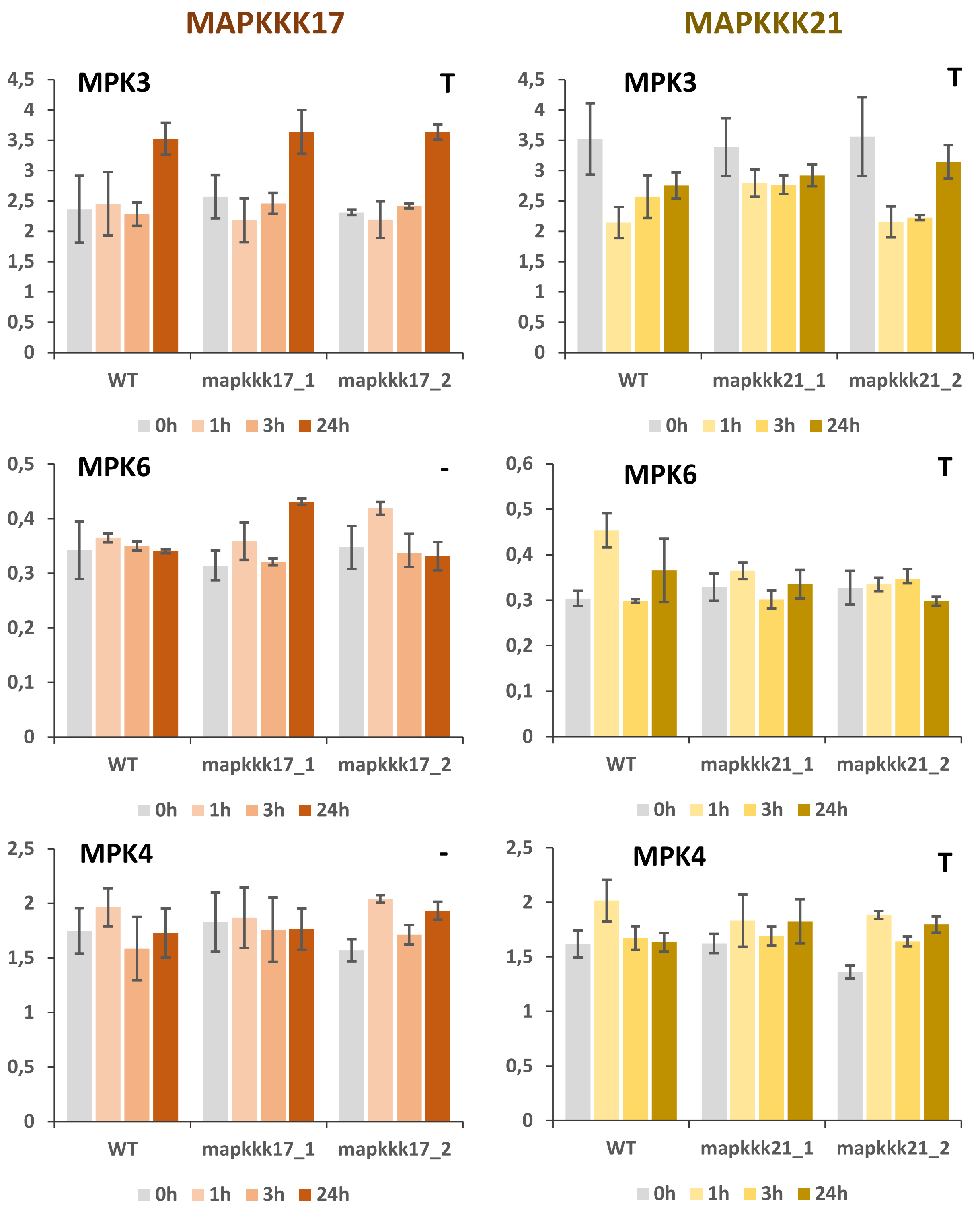

Suppl. Figure 4. Relative expression of *MPK3*, *MPK6*, and *MPK4* genes in different Arabidopsis backgrounds. Gene expression was quantified by RT-qPCR after 1h, 3h, and 24h of mite feeding on WT and T-DNA mutant lines. Data are means ± SE of three biological replicates. Significant factors (SF) indicate whether the two independent factors, T (treatment with mites) and G (genotype), and/or their interaction, I (T×G), were statistically significant (Two-way ANOVA, P<0.05, followed by Student-Newman-Keuls multiple comparisons test).
