# Supplementary material for: Opposite roles of MAPKKK17 and MAPKKK21 against Tetranychus urticae in Arabidopsis
Source: Front Plant Sci. 2022 Dec 7;13:1038866. doi: 10.3389/fpls.2022.1038866 (PMC9768502; doi:10.3389/fpls.2022.1038866)
Supplement: Supplementary Figure 5 — Western blot analysis of MAPK activation after 1h, 3h, and 24h of mite treatment in WT and T-DNA mutant lines. Flagellin treatment was used as a control of band size for MPK3/4/6. Activated MAPKs were detected by immunoblots using α-p44/42-ERK antibody. Equal loading is indicated by the Ponceau S staining of Rubisco. [file Image_5.pdf]

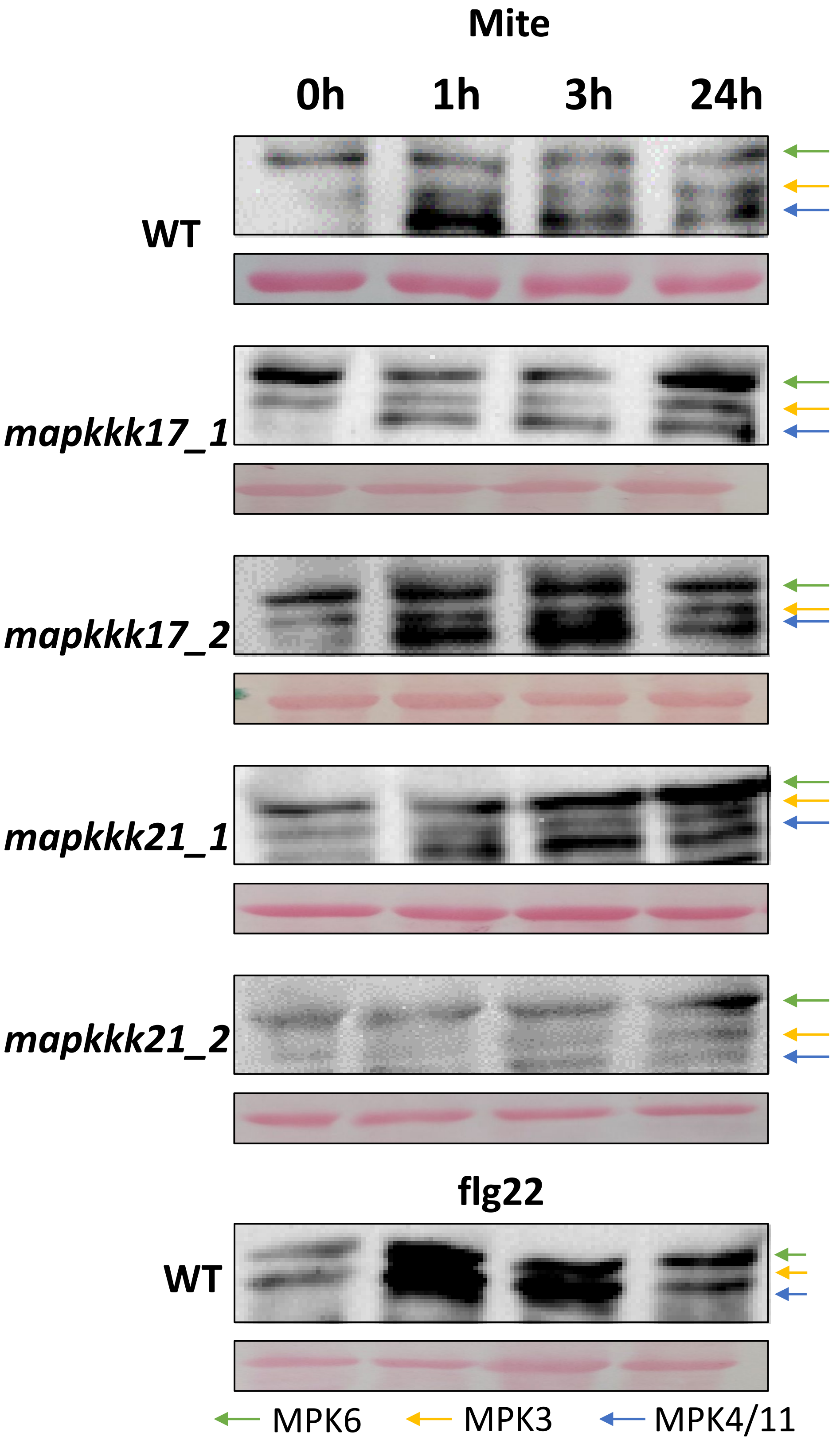

Suppl. Figure 5. Western blot analysis of MAPK activation after 1h, 3h, and 24h of flagellin (flg22) or mite treatment in WT and T-DNA mutant lines. Activated MAPKs were detected by immunoblots using α-p44/42-ERK antibody. Equal loading is indicated by the Ponceau S staining of Rubisco.
