# Supplementary material for: Opposite roles of MAPKKK17 and MAPKKK21 against Tetranychus urticae in Arabidopsis
Source: Front Plant Sci. 2022 Dec 7;13:1038866. doi: 10.3389/fpls.2022.1038866 (PMC9768502; doi:10.3389/fpls.2022.1038866)
Supplement: Supplementary Table 1 — Sequences of the oligonucleotides used in this work. [file Table_1.docx]

**Supplementary Table 1.** Sequences of oligonucleotide used primers (F: forward; R: reverse; LP: left primer; RP: right primer).

| **GENE ID** | **GENE NAME** | **Primer name** | **Primers 5´-3´** | **Purpose** |
| --- | --- | --- | --- | --- |
| *AT1G51660* | *MKK4* | 018804-LP | GCAACCAAAGGAACCTAAAGG | CONVENTIONAL PCR |
|  |  | 018804-RP | GAGGTTTCCTTTCCCTGTGAG |  |
| *AT1G01560* | *MPK11* | 049352-LP | TGCTCGAAATCAAAATGGAAC |  |
|  |  | 049352-RP | AATAAGACCACCTCAGCCAGAC |  |
| *AT2G32510* | *MAPKKK17* | 080309-LP | CCAAGAGAGAGTCAAACGGTG |  |
|  |  | 080309-RP | ATAACAGGCCAACAAACGTTG |  |
|  |  | 137069-LP | CTTGATGAATCGTCACATTGG |  |
|  |  | 137069-RP | ATGAAAGATGGACAGCGACAC |  |
| *AT4G36950* | *MAPKKK21* | 149019-LP | AGAGATTTTGAGCATGCATGC |  |
|  |  | 149019-RP | AGCTAAACTACCACCGGAAGC |  |
|  |  | 018714-LP | ATTCGCTGGTCGAACTACATG |  |
|  |  | 018714-RP | TGAGATGCTTTTGGATCATCC |  |
| *AT5G25760* | *Ubiquitin* | F-UBQ | GCTCTTATCAAAGGACCTTCGG | RT-qPCR |
|  |  | R-UBQ | CGAACTTGAGGAGGTTGCAAAG |  |
| *AT1G51660* | *MKK4* | F-MKK4 | CCTCTTCCTCTCCCACCTACTT |  |
|  |  | R-MKK4 | TCCGATACGGTTACCTCTCACT |  |
| *AT1G01560* | *MPK11* | F-MPK11 | GCTATCGACATTTGGTCTGTCG |  |
|  |  | R-MPK11 | TCAGTGATGAGTCTAAGCTGCTG |  |
| *AT2G32510* | *MAPKKK17* | F-MAPKKK17 | CGGATACAGAGGATCAGAAACC |  |
|  |  | R-MAPKKK17 | ACTTCACTACCCGAGTCTCGTC |  |
| *AT4G36950* | *MAPKKK21* | F-MAPKKK21 | GTTGTATATGGCACCGGAGTCT |  |
|  |  | R-MAPKKK21 | ATAACACCATCCTCCAAACACC |  |
| *AT1G64280* | *NPR1* | F-NPR1 | TGCATCAGAAGCAACTTTGG |  |
|  |  | R-NPR1 | GGCCTTTGAGAGAATGCTTG |  |
| *AT2G14610* | *PR1* | F-PR1 | TCAGTGAGACTCGGATGTGC |  |
|  |  | R-PR1 | CGTTCACATAATTCCCACGA |  |
| *AT1G32640* | *MYC2* | F-MYC2 | TCCGAGTCCGGTTCATTCT |  |
|  |  | R-MYC2 | TCTCGGGAGAAAGTGTTATTGAA |  |
| *AT5G24770* | *VSP2* | F-VSP2 | ATGCCAAAGGACTTGCCCTA |  |
|  |  | R-VSP2 | CGGGTCGGTCTTCTCTGTTC |  |
| [*AT3G45640*](https://www.arabidopsis.org/servlets/TairObject?id=37583&type=locus) | *MPK3* | F-MPK3 | GAATGATGAGCCAATCTGTCAA |  |
|  |  | R-MPK3 | CCGTATGTTGGATTGAGTGCTA |  |
| [*AT4G01370*](https://www.arabidopsis.org/servlets/TairObject?id=128006&type=locus) | *MPK4* | F-MPK4 | AGGATCTCCTGATGACTCAAGC |  |
|  |  | R-MPK4 | AGCAGCAAAGTTCTGTCTAGGG |  |
| [*AT2G43790*](https://www.arabidopsis.org/servlets/TairObject?id=32143&type=locus) | *MPK6* | F-MPK6 | TTAATGGACCGTAAGCCACTCT |  |
|  |  | R-MPK6 | ACTCGAGCTCTTCTTCTGATGG |  |
